# Progressive collapse potential of different types of irregular buildings located in diverse seismic sites

**DOI:** 10.1016/j.heliyon.2019.e01137

**Published:** 2019-01-16

**Authors:** Hamed Yavari, Mohammad Soheil Ghobadi, Mansoor Yakhchalian

**Affiliations:** Department of Civil Engineering, Faculty of Engineering and Technology, Imam Khomeini International University, PO Box 34149-16818, Qazvin, Iran

**Keywords:** Civil engineering, Structural engineering

## Abstract

This paper evaluates the effects of severity of Torsional Irregularity (TI) and In-plane Discontinuity in Vertical Lateral force-resisting element Irregularity (IDVLI) together with seismic strength of the building on the progressive collapse potential of steel Special Moment-Resisting Frames (steel SMRFs), which were designed based on common seismic codes. In order to investigate the progressive collapse potential according to GSA 2013 guidelines, an interior or exterior column is removed in 3D modeled building using nonlinear dynamic analysis. Various TIs by defining the ratio of maximum relative lateral displacement of the story to average relative lateral displacement of the story between 1 to 1.6 and IDVLIs by disconnecting one or two columns in the first and second stories are selected. Buildings are 3, 6 and 9 stories high, and Los Angeles, California andGeorgia sites with high, moderate and low levels of seismicity, respectively, are considered. All corresponding buildings have similar seismic mass and are designed for approximately equal values of earthquake base shear, so the comparison process can be possible due to the comparison of equivalent-designed buildings. Gravity and seismic loads of buildings are applied based on ASCE 7-05, and steel design is carried out based on AISC 2010. The results show that buildings designed with greater TI have greater resistance to the progressive collapse phenomenon. Furthermore, buildings in a site with higher seismicity level have less progressive collapse potential. In IDVLI, the buildings located in a site with low seismicity are always rejected against progressive failure based on GSA 2013, whereas buildings located in a site with high seismicity are always acceptable. In addition, in a system with IDVLI, the scenario of external column removal always creates more critical conditions. Results toward the combined effects of irregularity and seismicity level of sites are presented.

## 1 Introduction

**Fig. 1:**
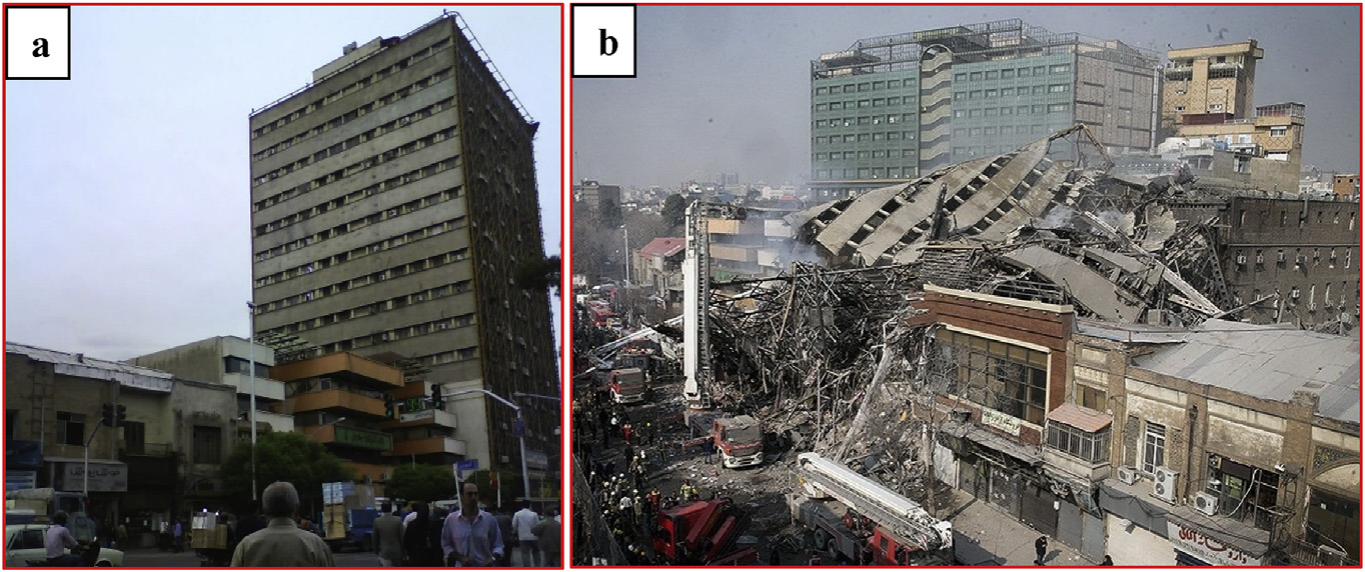
The Plasco building in Tehran (a) before progressive collapse [5] (b) after progressive collapse [6].

Attention to the issue of progressive collapse was made for the first time in the engineering community due to occurrence of a local collapse in the 22-story Ronan Point building [1] in London, which happened in 1968 due to gas leakage on the eighteenth story. Also, the bombing of the Alfred P. Murrah building [2] in Oklahoma in 1995 was one of the largest terrorist incidents that led to a progressive collapse phenomenon, causing a loss of 652 million dollars. In addition, the September 11 incident in the World Trade Towers [3, 4] in 2001, as an effective event, sparked further attention to the issue of progressive collapse. Progressive collapse mostly begins due to factors such as explosion or fire, and it continues due to chain collapse of structural members up to complete destruction of building, and its progress cannot be controlled. The progressive failure in the Plasco building in Tehran [5, 6] caused by fire was another tragedy, resulting in the death of twenty firefighters (Fig. 1).

Standards for the design and control of buildings against progressive failure include the guidelines of the US Department of Defense (DoD) [7] and the US General Services Administration (GSA) [8]. Different methods for controlling and preventing progressive collapse are presented in these standards. Most of these methods are based on the chain strength of the existing members to transfer the force after the removal of an element in the building. These codes have been revised over time [9, 10, 11], and more up-to-date guidelines have been added to them. In the 2013 editions of GSA [9] and Unified Facilities Criteria (UFC)-DoD [11], three-dimensional (3D) analysis methods were added and required. In addition, the combination of different loads for progressive failure was considered, and detailed information was provided for various linear and nonlinear analyses of progressive collapse.

Proper arrangement of columns, more redundancy in the building, use of beam-to-column connections with the possibility of transferring the axial forces resulting from the removal of the destructed column and higher energy absorption capacity in the building are some concepts of improving the structural resistance to the progressive collapse phenomenon. Several investigations have been conducted on progressive collapse control in steel buildings, and the cases related to the subject of this research are referred here. Hayes et al. [12] seismically retrofitted an attacked building in 1995 and investigated whether “seismic retrofitting of the building can improve the resistance of the building to progressive collapse.” They concluded that retrofitting the peripheral members of the building would increase the resistance to progressive collapse well. Kim et al. [13], based on both the GSA and DoD guidelines, investigated the strength of steel buildings against progressive failure by linear and nonlinear analytical methods. They reported that linear methods of the codes have conservative results, and nonlinear dynamic analysis is a more appropriate tool for controlling the progressive failure phenomenon in complex situations. Karimiyan et al. [14, 15] examined the progressive failure in two symmetric and asymmetric reinforced concrete buildings. The severity of earthquake and eccentricity of the rigidity centroid compared to mass centroid were the variables of the study. They concluded that regardless of the severity of the earthquake, there is a progressive failure pattern at the place of mass accumulation, and the amount of eccentricity in plan changes the progressive collapse pattern. Tavakkoli and Alashti [16] investigated whether earthquake-resistant buildings could resist progressive failure. For this purpose, they selected two types of 3D and 2D analyses, two heights of 5 and 15 stories, and two spans of 4 and 6 m as variables and used UFC-DoD to investigate the progressive failure. The results showed that 3D models exhibit greater strength of the building. In addition, increasing the height of the building and the number of spans would increase resistance against progressive collapse. Meanwhile, they reported that earthquake-resistant buildings do not have the potential for progressive failure and are strong enough. Kordbagh and Mohammadi [17] investigated the effect of the building height and the seismicity of site on the resistance to progressive collapse. They reported that increasing the height would increase resistance to progressive failure, and higher seismicity of the site would also increase structural strength. Koofild and Adeli [18] examined the effect of geometric irregularities in plan and height in moment-resisting frame buildings including concentrically and eccentrically braced frames under the influence of explosion and progressive collapse. They concluded that the strength of concentrically braced frames is greater than those of other systems, and irregularity has a negative effect on the progressive collapse resistance of buildings. Ebrahimi et al. [19] investigated the effect of plan irregularity on the progressive collapse resistance of four steel buildings located in sites with soil classes C and E. The results showed that the irregular building located in the site with soil class C had the worst conditions, and in the case of the buildings located in a site with soil class E the demand to capacity ratio (D/C) of column in the irregular building was twice that of the regular building. Kim and Hung [20] compared the progressive failure potential of an irregular 30-story tower with an equivalent regular tower. The results of their study showed that resistance to the progressive failure potential of the irregular tower was greater than that of the regular tower. Moreover, the location of the column removal in the irregular building had a significant effect on the results. Khandelwal et al. [21] investigated the resistance to progressive collapse in Special Concentrically Braced Frames (SCBFs) and Eccentrically Braced Frames (EBFs). The results of their study showed that the EBF system is less vulnerable to progressive collapse than the SCBF system.

This research aims to investigate the progressive collapse potential of 3D irregular buildings with different scenarios for removing external and internal columns. For this purpose, different severities of Torsional Irregularity (TI) and different modes of In-plane Discontinuity in Lateral force-resisting system Irregularity (IDVLI), which can be one or two columns cut-off in the first or the second story, are considered. Buildings have 3, 6 and 9 stories high with steel Special Moment-Resisting Frame (steel SMRF) system. The plans of buildings selected for the case of TI have the same areas, with different TI severities and shapes. However, one plan is selected for the buildings with IDVLI, and different modes of column cut-off in the first and second stories are considered. To design these buildings, three sites with different seismicity levels located in Los Angeles (LAS), California (CS) and Georgia (GS) are selected, and the parameters of the design spectrum for each of the three sites are extracted from the USGS [22] website. These sites respectively represent high, moderate, and low seismic hazard levels. ASCE 7-05 [23] is used to determine the gravity and seismic loads required for designing the buildings. 3D models of all the buildings are created using ETABS v15.1 [24], and the structural designs are performed in accordance with AISC 360-10 [25]. Then, with the help of a converter software [26], the structural models are taken from ETABS v15.1 [24] software to SAP2000 v17.1.1 [27] software, and nonlinear dynamic analyses are carried out based on GSA 2013 [9] on each one of the buildings for different column removal scenarios. Indeed, this research compares the equivalent-designed buildings with approximately the same base shear values, which have various intensities of TI and different modes of IDVLI in height. The variations of the studied buildings are in terms of seismicity level, height, and type of irregularity. Few research studies evaluate the effect of seismicity and irregularity together. In results, time histories of vertical displacement of the node located above the removed column in the buildings with various intensities of TI and different modes of IDVLI including one or two columns cut-off in the first or second story are presented. Then by comparing them with each other, in terms of resistance to progressive collapse and scenarios of removing internal columns and external columns conclusions are drawn.

## 2 Methodology

### 2.1 Analytical methods of progressive collapse evaluation

In order to study the progressive collapse potential of buildings, the alternative load path method has been introduced in the GSA Code 2013 [9]. In this method, a column of the building is lost under unconventional loads such as explosion or collision, and the other structural members must be able to prevent the collapse in the building. Three analytical linear static, nonlinear static and nonlinear dynamic methods are proposed in this guideline. Certainly, for the study of TI and IDVLI in height, the models of buildings must be three-dimensional, and the analytical method must be precise. In the nonlinear static analysis, to account for dynamic effects, in the spans adjacent to the column removal location, a coefficient called the "omega enhancement coefficient" must be used, which is equal to two. In general, nonlinear static analysis is less accurate and more conservative than nonlinear dynamic analysis. Nonlinear dynamic analysis is the most accurate type of analysis and has the highest sensitivity to dynamic parameters. In nonlinear dynamic analysis second order effects i.e., geometrical nonlinearity in the large displacement domain, are accounted for in the analysis to have real vertical deformation. In fact, the reason for the higher accuracy of nonlinear dynamic analysis compared to other methods for analyzing the progressive collapse phenomenon is that this method is closer to reality. After progressive collapse analysis, if the building exceeds the Collapse Prevention (CP) performance level [28], it will be rejected, whereas if the plastic hinges of the building remain within CP, LS (Life Safety), and IO (Immediate Occupancy) performance levels [28] the building will be stable and acceptable. In order to find the most critical condition of progressive collapse, different scenarios of interior and exterior column removal must be examined.

In this research, the nonlinear dynamic analysis method was selected to evaluate the buildings, and its stages were as follows:

1- Selecting the intended column and obtaining the internal forces of the column, where the following gravity load combination is used to calculate the internal forces [9]:

(1) G_ND_ = 1.2D + (0.5L or 0.2S)

where $G_{ND}$ is gravity loads for nonlinear dynamic analysis, D is dead load, L is Live load, and S is Snow load.

2- Assigning the internal forces in the node located at the top of the removed column as the reaction forces, and performing nonlinear static analysis until the gravity loads and applied reaction forces achieve the prerequisite static equilibrium. If the column of a story other than the first story is selected for removal, reaction forces should be generally applied not only to the node located at the top of the column to be removed, but also to the node located at the bottom of that column.
3- After the structural equilibrium is achieved, the reactions are eliminated in a fraction of a second and the nonlinear dynamic analysis is performed. According to GSA 2013 [9], the time needed to remove the reaction forces, or in other words, to remove the damaged column, must be less than $\frac{1}{10}$ of the first period of the vertical vibrational mode of the building with the removed column. In this research, the removal time of the column is assumed equal to $\frac{1}{15}$ of the aforementioned period.

**Fig. 2:**
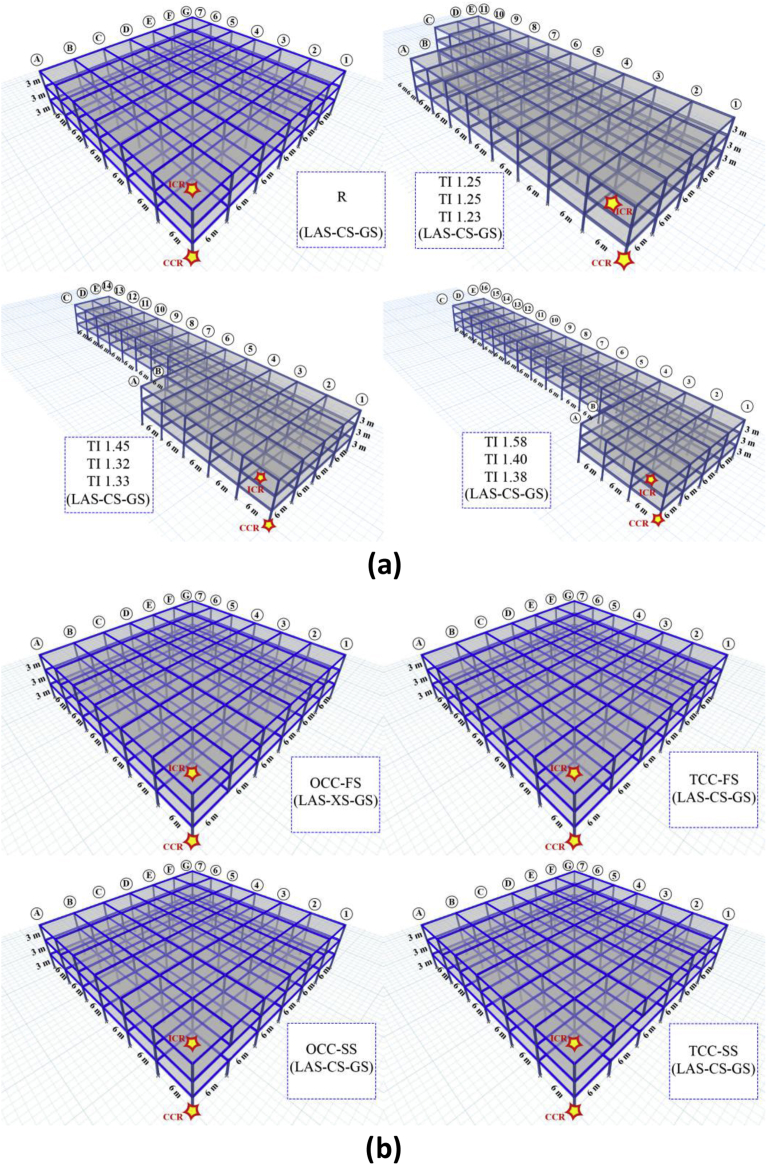
Schematic view of different scenarios of removing internal and external columns in 3D structural models: (a) buildings with TI and (b) IDVLI.

An example of different scenarios for removing internal and external columns three-dimensionally in a 3-story building with TI and IDVLI is shown in Fig. 2. In this paper, only IDVLI cas es were considered that are due to column cut-offs located at building perimeter. Column cut-off in the perimeter of buildings has architectural benefits in large entrance of hotels, great residential buildings, commercial complexes, etc. For the inner column cut-off examples another research work should be done. In total, 72 buildings were designed and evaluated. For each building, more than two nonlinear dynamic analyses were conducted for the internal and external column removal scenarios, resulting in more than 144 nonlinear dynamic analyses. For this reason, the most critical exterior and interior scenarios were selected and reported to achieve the most critical condition of progressive collapse. Indeed, in each exterior and interior scenario, some choices were tested and the most critical choice was reported. Columns with larger demand to capacity ratio or columns adjacent to mentioned columns had greater chance of more critical progressive collapse situation with greater vertical displacement at point of removed column. In IDVLI cases, columns near the cut-off columns were removed to observe the critical progressive collapse potential in both exterior and interior scenarios. The duration of nonlinear dynamic analyses for the aforementioned models was considered to be five seconds.

## 3 Model

### 3.1 Building modeling descriptions

The building models studied in this research were 3-, 6-, and 9-story steel buildings with story height of three meters (see Fig. 2) with residential use for the three previously mentioned sites, i.e., LAS, CS and GS, representing high, moderate and low seismicity, respectively. With regard to Fig. 2 the buildings have a square (regular (R)) or L-shaped (irregular) plan with a same span length of 6 meters. 3D models of this figure show buildings with TI and IDVLI. The plan area of all buildings is equal, and therefore the base shear values obtained are almost the same. The floor system is one-way ribbed slab, and rib directions in adjacent spans are perpendicular to each other. In Table 1, the assumed gravity loads are presented.

**Table 1:** Gravity loads applied on the building.

Row	The type of loads	Dead load	Live load
1	Floors	5.76 $\left(\frac{kN}{m2}\right)$	2 $\;\left(\frac{kN}{m2}\right)$
2	Roof	5.25 $\left(\frac{kN}{m2}\right)$	1 $\;\;\left(\frac{kN}{m2}\right)$
3	Cladding	6.65 $\left(\frac{kN}{m}\right)$	---
4	Parapets	2.6 $\left(\frac{kN}{m}\right)$	---

**Fig. 3:**
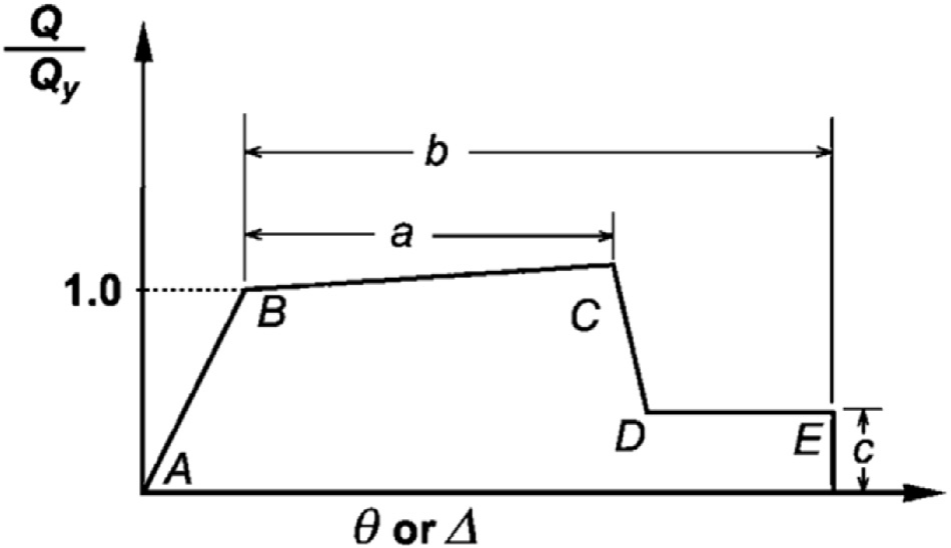
Generalized force-deformation relation for steel elements [28].

A992 Steel [29] with yield stress of ${\text{F}}_{y}=50\;\text{ksi}$ was used for design of buildings. The assumption of concentrated plasticity in plastic hinges [28] of beams and columns was considered for including nonlinear behavior of materials. The plastic hinge characteristics [28] were defined in the SAP2000 [27] software using geometric data and material specifications. All plastic hinges are modeled by the force-deformation relation of Fig. 3 and each hinge specifications are adopted from Chapter 5 of ASCE 41-06 [28]. Most of the nonlinear modeling criteria from Chapter 5 of ASCE 41-06 [28] are explicitly adopted in GSA 2013 [9]. Based on tables of ASCE 41-06 [28], steel material properties and beam/column section geometric characteristics, the hinge behavioral diagram is determined for each section.

The locations of the plastic hinges in the beams and columns were considered at the vicinity of the beam-column node, and another hinge was also foreseen in the middle of each beam. The 72 buildings were designed by using 3D models and the geometric characteristics of obtained sections for their beams and columns are provided in the appendix file of this article, due to high volume of information. Geometric nonlinearity was considered in the progressive collapse analysis by SAP 2000 [27] program to achieve the real value of vertical displacement at the node that column is removed under it.

### 3.2 Seismic specifications of buildings

Based on the latitude and longitude of the sites, including LAS (longitude: −118.162°, latitude: 33.996°), CS (longitude: −120° latitude: 36.7°) and GS (longitude: −83.5°, latitude: 32.985°), seismic parameters of the sites were extracted from the USGS website [22]. Seismic loading was carried out according to ASCE 7-05 [23], and structural design was conducted based on AISC 360-10 [25] considering the steel SMRF system as the lateral force-resisting system of the buildings. According to ASCE 7-05 [23], the base shear coefficient, ${\text{C}}_{\text{s}}$, is obtained according to the following relations:

(2) $${\text{C}}_{\text{s}}=\frac{{\text{S}}_{\text{DS}}}{\frac{\text{R}}{{\text{I}}_{\text{e}}}}\;\;\text{T}<{\text{T}}_{\text{s}}$$

(3) $${\text{C}}_{\text{s}}=\frac{{\text{S}}_{\text{D}1}}{\text{T}\left(\frac{\text{R}}{{\text{I}}_{\text{e}}}\right)}\;\;{\text{T}}_{\text{s}}<\text{T}<{\text{T}}_{\text{L}}$$

(4) $${\text{C}}_{\text{s}}=\frac{{\text{S}}_{\text{D}1\;}.{\text{T}}_{\text{L}}}{{\text{T}}^{2}\left(\frac{\text{R}}{{\text{I}}_{\text{e}}}\right)}\;\;\text{T}>{\text{T}}_{\text{L}}$$

In the above relations, the calculated values of ${\text{C}}_{\text{s}}$ must not be less than the value obtained using the following equation:

(5) $${\text{C}}_{\text{s}}=0.044\;{\text{S}}_{\text{DS}}\;I_{\text{e}}\geq 0.01$$

Moreover, in areas where ${\text{S}}_{1\;}$is equal to or greater than 0.6g, ${\text{C}}_{\text{s}}$ must not be considered less than the value obtained from the following equation:

(6) $${\text{C}}_{\text{s}}=\frac{0.5{\text{S}}_{1}}{\left(\frac{\text{R}}{{\text{I}}_{\text{e}}}\right)}$$

The summary of calculations of the base shear coefficient for all buildings with the steel SMRF system, according to ASCE 7-05 [23], for the three sites, i.e., LAS, CS and GS is presented in Table 2. Symbols presented in Eqs. (2), (3), (4), (5), and (6) and Table 2 are defined as follows:

$S_{DS}$ = design, 5 percent damped, spectral response acceleration parameter at short periods as defined in ASCE 7-05 [23]
$S_{D1}$ = design, 5 percent damped, spectral response acceleration parameter at a period of 1 s as defined in ASCE 7-05 [23]
$S_{1}$ = mapped MCE, 5 percent damped, spectral response acceleration parameter at a period of 1 s as defined in ASCE7-05 [23]
$S_{s}$ = mapped MCE, 5 percent damped, spectral response acceleration parameter at short periods as defined in ASCE 7-05 [23]
R = response modification coefficient as given in ASCE 7-05 [23]
$I_{e}$ = the importance factor as prescribed in ASCE 7-05 [23]
T = the fundamental period of the building
$T_{L}$ = long-period transition period as defined in ASCE 7-05 [23]
$T_{s}=\;S_{D1}/S_{DS}$
$F_{a}$ = short-period site coefficient (at 0.2 s-period)
$F_{v}$ = long-period site coefficient (at 1.0 s-period)
k = distribution exponent given in ASCE 7-05 [23]

**Table 2:** Calculation of base shear coefficients for the 3-, 6-, and 9-story structures.

Parameters	LAS			CS			GS		
	3-story	6-story	9-story	3-story	6-story	9-story	3-story	6-story	9-story
Occupancy category	II	II	II	II	II	II	II	II	II
$H_{n}\;\left(m\right)$	9	18	27	9	18	27	9	18	27
Site Class	D	D	D	D	D	D	D	D	D
$s_{s}$	1.74	1.74	1.74	0.82	0.82	0.82	0.40	0.40	0.401
$s_{1}$	0.608	0.608	0.608	0.29	0.29	0.29	0.12	0.12	0.122
R	8	8	8	8	8	8	8	8	8
$I_{e}$	1	1	1	1	1	1	1	1	1
$F_{a}$	1	1	1	1.172	1.172	1.172	1.48	1.48	1.479
$F_{v}$	1.5	1.5	1.5	1.818	1.818	1.818	2.313	2.313	2.313
$T_{L}$	8	8	8	8	8	8	8	8	8
T (s)	0.588	1.024	1.416	0.588	1.024	1.416	0.63	1.097	1.51677
k	1.044	1.262	1.458	1.044	1.2618	1.458	1.065	1.298	1.5084
$C_{s}$	0.129	0.074	0.054	0.075	0.043	0.031	0.056	0.032	0.023

**Fig. 4:**
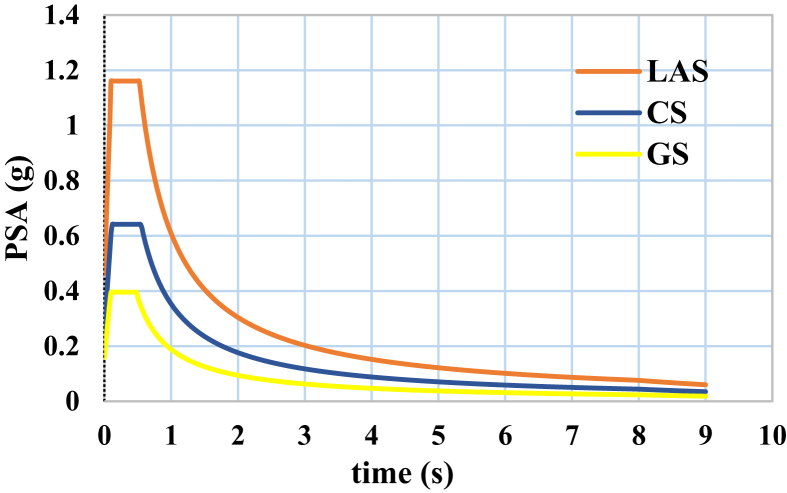
ASCE 7-05 design spectra for the three considered sites (i.e., LAS, CS, GS).

Seismic analysis of buildings was based on the modal response spectrum analysis method, and both controls of strength and structural drift have been performed. Design spectra of the considered sites are shown in Fig. 4. In 3-story buildings located in the sites with high and medium hazard levels, drift control was dominant in the structural design. However, in 3-story buildings located in the site with low hazard level, strength control of members was dominant in the design process. In 6- and 9-story buildings located in all the three sites with high, medium, and low hazard levels, the design was only governed by drift control. In the design process, it was attempted to design buildings accurately and close to allowed code limits, to be able to precisely evaluate the effects of the two previously mentioned types of irregularity and the seismicity level of site on the progressive collapse behavior of buildings. All deigned profile sections of beams and columns are presented in supplementary excel file (designed profile section of buildings.xlsx), which is associated with this paper in journal website. In this file each frame section is introduced with its numbering and location.

### 3.3 Different irregularities definition

According to ASCE 7-05 [23], in order to control the TI for the building, the ratio of maximum relative displacement at one end of the building plan in each story under the influence of seismic lateral forces to the average of relative displacements at two ends of the building plan determines the intensity of TI. If the intensity ratio is greater than 1.20, the building has TI, and if the ratio is greater than 1.40, the TI is severe. In this research, the maximum intensity of TI of different stories of each supposed building is selected as the index of intensity of TI, and the building is named with that. When a component of the lateral force-resisting system is discontinued in height, this discontinuity causes overturning moments on beams, slabs, columns and supporting walls, and is classified as IDVLI. In other words, IDVLI can emerge due to column cut-off in architectural voids of structures.

## 4 Results and discussion

**Fig. 5:**
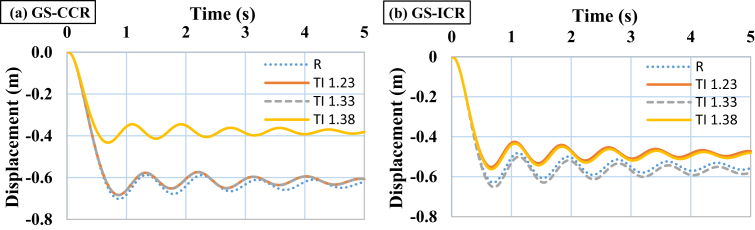
Time histories of vertical displacement of the node at the top of the removed column in the 3-story structures with different intensities of TI, (a) GS with corner column removal scenario (b) GS with internal column removal scenario.

**Fig. 6:**
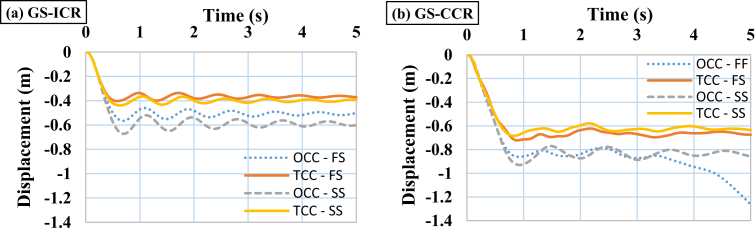
Time histories of vertical displacement of the node at the top of the removed column in the 3-story structures with IDVLI due to column cut-off in the first and second story (a) GS with internal column removal scenario (b) GS with corner column removal scenario.

When performing progressive failure analyses, different scenarios of removing columns (internal and external columns) in 3-, 6-, and 9-story buildings with various intensities of TI and various modes of IDVLI were evaluated. In the figures and tables presented in this section, ICR denotes that internal column is removed, whereas CCR denotes that corner column is removed. The results were acquired in terms of vertical displacement time histories of the nodes at the top of the removed columns. As an example, the obtained graphs of the vertical displacement time histories in the 3-story buildings located in GS with TI and IDVLI, respectively, are shown in Figs. 5 and 6. In Fig. 6, the results corresponding to various modes of IDVLI including one column cut-off in the first story (OCC-FS), two columns cut-off in the first story (TCC-FS), one column cut-off in the second story (OCC-SS) and two columns cut-off in the second story (TCC-SS) are shown. Inherently, there is a significant relation between seismic hazard level and the amount of formation of plastic hinges in progressive collapse analysis of the buildings. However, despite the different intensities of TI and various modes of column cut-off in first and second stories in equivalent buildings (with same base shear) and different scenarios of removing columns, the significant relation among acquired patterns of buildings with TI and IDVLI analyses must be determined. In the following, each one of aforementioned irregularities' effects on the progressive collapse potential is interpreted.

### 4.1 Progressive collapse analyses of buildings with TI

Table 3 shows the matrix of output information obtained from progressive failure analyses of the considered buildings. In this table, the values of vertical displacement in the node at the top of the removed column, respectively, for 3-, 6- and 9-story buildings designed for all the sites are presented for both scenarios of removing columns. It should be mentioned that T is the elastic period of the first lateral mode of structure before column removal or the elastic period of the first vertical mode of structure with a removed column. As it is observed in Table 3, for the regular 3-story building located in LAS with high seismicity, the value of vertical displacement of the removed external column has reduced 31% and 54% compared to those of buildings located in sites with medium (LAS) and low (GS) seismicity, respectively. These percentages are respectively 25% and 33% for the scenarios of removing the internal column. In addition, in irregular buildings with maximum TI intensity, in the 3-story building located in Los Angles, the vertical displacement of the removed column has reduced 81% and 84% compared to LAS and GS, respectively. Generally, by observing the results presented in Table 3, it can be stated that the more irregular the building is and the higher the seismicity level of the site is, the greater is the resistance to progressive collapse. For instance, considering the scenario of removing the external column, the 3-story building located in LAS with maximum irregularity intensity shows approximately 6 cm of vertical displacement in the node at the top of the removed column, whereas the regular building located in GS shows approximately 56 cm vertical displacement. Meanwhile, regardless of the scenarios of removing internal and external columns, irregular buildings have better resistance to progressive failure than regular buildings. In other words, irregular buildings compared to regular buildings that are equivalent in terms of design base shear have better resistance to progressive failure. This is because considering the design requirements for TI in buildings leads to larger size of sections used in beams and columns. It is noteworthy that these behaviors are also observed in taller buildings.

**Table 3:** Matrix of parameters obtained from the progressive collapse analyses of buildings with and without TI.

Number of stories	Type of site	Building	T (s)	First vertical Mode (ICR)	First vertical Mode (CCR)	Δz (m) (CCR)	Performance level	Δz (m) (ICR)	Performance level
3-story	LAS	R	1.466	mode = 4 T = 0.68	mode = 4 T = 0.7	−0.268	LS	−0.366	LS
		TI 1.25	1.271	mode = 4 T = 0.51	mode = 4 T = 0.56	−0.092	IO	−0.112	IO
		TI 1.45	1.228	mode = 4 T = 0.48	mode = 4 T = 0.54	−0.075	IO	−0.089	IO
		TI 1.58	1.073	mode = 4 T = 0.43	mode = 4 T = 0.48	−0.059	-	−0.062	IO
	CS	R	1.788	mode = 4 T = 0.76	mode = 4 T = 0.79	−0.418	LS	−0.472	LS
		TI 1.25	1.781	mode = 4 T = 0.75	mode = 4 T = 0.8	−0.4	LS	−0.457	LS
		TI 1.32	1.72	mode = 4 T = 0.74	mode = 4 T = 0.73	−0.29	LS	−0.444	LS
		TI 1.40	1.665	mode = 4 T = 0.67	mode = 4 T = 0.77	−0.318	LS	−0.318	LS
	GS	R	2.038	mode = 4 T = 0.8	mode = 4 T = 0.89	−0.622	CP	−0.557	LS
		TI 1.23	1.871	mode = 4 T = 0.76	mode = 4 T = 0.86	−0.606	CP	−0.472	LS
		TI 1.33	1.837	mode = 4 T = 0.8	mode = 4 T = 0.86	−0.6071	CP	−0.5795	CP
		TI 1.38	1.741	mode = 4 T = 0.76	mode = 4 T = 0.76	−0.382	LS	−0.48	LS
6-story	LAS	R	2.155	mode = 9 T = 0.44	mode = 7 T = 0.48	−0.069	IO	−0.07	IO
		TI 1.26	1.869	mode = 9 T = 0.39	mode = 7 T = 0.42	−0.051	-	−0.052	-
		TI 1.45	1.725	mode = 10 T = 0.34	mode = 7 T = 0.38	−0.04	-	−0.0403	-
		TI 1.58	1.711	mode = 10 T = 0.35	mode = 7 T = 0.38	−0.04	-	−0.041	-
	CS	R	3.502	mode = 9 T = 0.68	mode = 9 T = 0.72	−0.318	LS	−0.375	LS
		TI 1.25	3.132	mode = 7 T = 0.66	mode = 7 T = 0.69	−0.307	LS	−0.34	LS
		TI 1.42	2.935	mode = 7 T = 0.63	mode = 7 T = 0.66	−0.25	LS	−0.278	LS
		TI 1.46	2.928	mode = 7 T = 0.62	mode = 7 T = 0.65	−0.236	LS	−0.265	LS
	GS	R	4.037	mode = 7 T = 0.84	mode = 7 T = 0.83	−0.594	CP	−0.685	CP
		TI 1.25	3.835	mode = 8 T = 0.78	mode = 7 T = 0.83	−0.626	CP	−0.548	CP
		TI 1.36	3.616	mode = 7 T = 0.75	mode = 7 T = 0.79	−0.532	CP	−0.498	LS
		TI 1.40	3.267	mode = 8 T = 0.67	mode = 7 T = 0.7	−0.323	LS	−0.369	LS
9-story	LAS	R	2.94	mode = 10 T = 0.45	mode = 10 T = 0.46	−0.068	IO	−0.079	IO
		TI 1.26	2.743	mode = 11 T = 0.4	mode = 10 T = 0.42	−0.056	-	−0.058	-
		TI 1.45	2.995	mode = 10 T = 0.4	mode = 10 T = 0.4	−0.051	-	−0.05	-
		TI 1.60	2.71	mode = 11 T = 0.38	mode = 10 T = 0.41	−0.052	-	−0.052	-
	CS	R	5.024	mode = 12 T = 0.67	mode = 12 T = 0.69	−0.34	LS	−0.369	LS
		TI 1.27	5.363	mode = 12 T = 0.66	mode = 10 T = 0.71	−0.347	LS	−0.349	LS
		TI 1.49	4.75	mode = 10 T = 0.67	mode = 10 T = 0.67	−0.294	LS	−0.36	LS
		TI 1.53	4.51	mode = 10 T = 0.62	mode = 10 T = 0.63	−0.23	LS	−0.29	LS
	GS	R	6.16	mode = 12 T = 0.77	mode = 10 T = 0.85	−0.565	CP	−0.556	CP
		TI 1.24	6.058	mode = 12 T = 0.76	mode = 10 T = 0.82	−0.427	LS	−0.535	CP
		TI 1.33	5.742	mode = 12 T = 0.73	mode = 10 T = 0.76	−0.369	LS	−0.477	LS
		TI 1.43	5.603	mode = 12 T = 0.72	mode = 10 T = 0.74	−0.34	LS	−0.454	LS

According to Table 3, buildings located in GS show the highest vertical displacements and buildings located in LAS with high seismicity show the lowest vertical displacements compared to the other buildings. In addition, a range for determining the performance level of buildings was acquired based on vertical displacements obtained from nonlinear dynamic analyses in the studied buildings. On the other hand, the performance level of formed plastic hinges of all buildings were checked at the end of the progressive collapse analysis and it was specified that building enter the determined performance level (IO, LS, or CP) [28]. Table 3 shows performance levels and vertical displacements in each progressive collapse analysis for exterior and interior analyses. If the vertical displacement at the top of removed column is in the range of 6 to 11 cm, the performance level of the building is in the IO range and this range takes place for buildings located in a site with a given seismic hazard level and given height. On the other hand, considering the length of span equal (L) to 6 m, if the vertical displacement of node at the removed column is in the range of (L/100) to (L/60) the structure performance level is in the IO range. Similarly, If the vertical displacement at the top of removed column is between (L/24) to (L/12), the performance level of the building is in the LS range given a seismicity level and height. Furthermore, if it is greater than (L/11), the performance level is in the CP range. It should be noted that this pattern existed almost in all the buildings studied and can be a convenient criterion for determining the performance level of building based on vertical displacement of the node at top of destructed column.

**Fig. 7:**
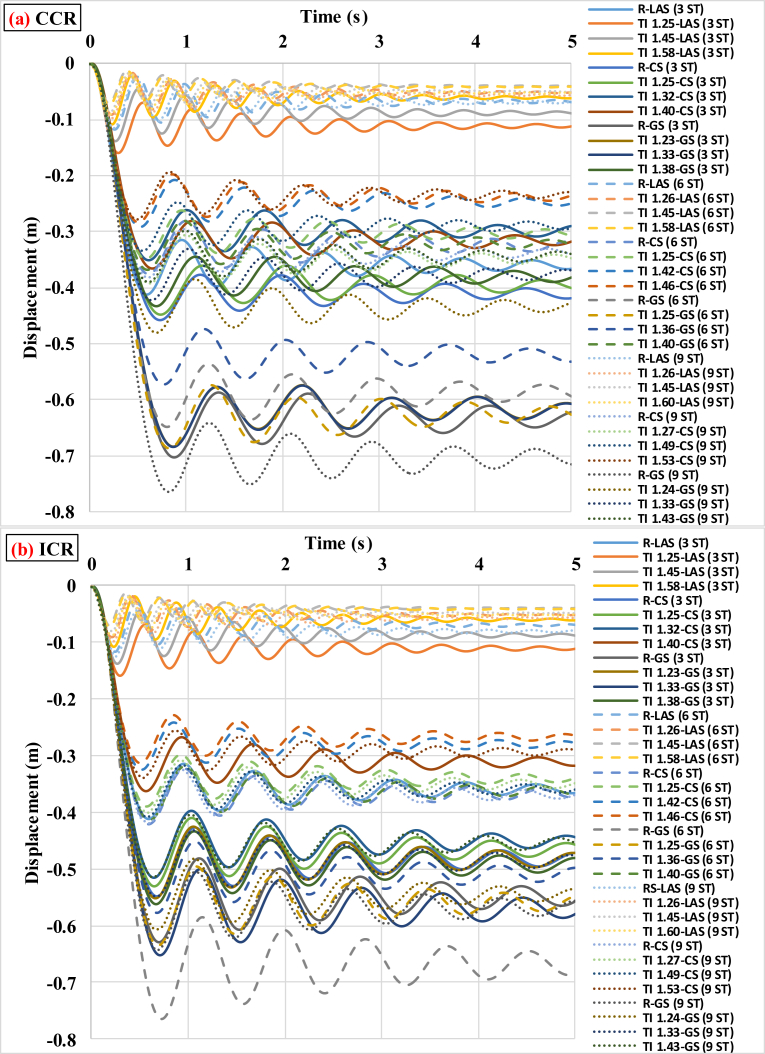
Vertical displacement histories of the 3-, 6- and 9-story buildings under different cases of TI assuming (a) CCR, (b) ICR.

Based on the data obtained from the progressive collapse analyses of buildings with TI, no accurate pattern was found for the criticality of scenarios of removing external and internal columns. On the other hand, in different sites and buildings with different severities of irregularity, both the scenarios of removing internal and external columns must be controlled. However, the number of buildings in which the scenario of removing the internal column led to greater vertical displacement was greater. Considering Table 3, in the 3-story buildings located in LAS, by increase in the values of TI intensity from the regular building to the buildings with the TI intensities of 1.25, 1.43 and 1.58, in the time of removing corner columns, values of vertical displacement are respectively reduced from −0.268 m to −0.092, −0.075 and −0.059 m. Therefore, increase in TI intensity to 1.58 compared with regular building leads to 78% decrease in displacement caused by progressive failure. The time histories of vertical displacements in all the studied buildings under TI located in different sites are shown in Fig. 7. In this figure, ST denotes story. In other words, 3 ST, 6 ST and 9 ST represent 3-, 6- and 9-story buildings, respectively.

As well, in the 6-story buildings located in LAS, by increase in values of TI intensity from the regular building to the buildings with TI intensities of 1.26, 1.45 and 1.58, in time of removing corner columns, the values of vertical displacement are respectively equal to −0.0689, −0.0513, and −0.04 m. Therefore, increase in TI intensity to 1.58 compared with the regular building leads to almost 42% decrease in the displacement caused by progressive failure. According to the above discussion, concern for progressive failure in cities with lower seismic hazard is more serious. Meanwhile, regular buildings, which mostly are representative of urban residential buildings, are exposed to more hazard due to progressive collapse phenomenon.

Table 4 indicates the number of hinges exceeding the CP level after progressive failure analyses. If in the building the plastic hinge exceeds the CP level, the building is rejected, and progressive failure will lead to its demolition. According to the aforementioned table, in 3-story buildings, the regular building and irregular buildings with TI intensities up to 1.33 located in the site with low seismicity level were rejected. In addition, in the case of 6-story buildings a similar trend was observed. Clearly, it can be found that increase in height, increase in base shear coefficient, and increase in TI strengthen the resistance to progressive collapse. In fact, all the buildings located in the site with high seismicity (LAS) showed good resistance to progressive failure. If a parameter is defined as the product of these three factors, it can specify the possibility of collapse in progressive failure. The failure parameter γ is defined as follows:

(7) γ = base shear coefficient × TI intensity × number of stories of building

**Table 4:** The number of plastic hinges exceeding the CP mode and the acceptance or rejection of progressive collapse analysis under TI.

Number of floors	Type of site	Building	Base shear coefficient	Effective seismic mass	Base shear	Number of plastic hinges exceeding CP	Number of plastic hinges exceeding CP	Structural condition after progressive collapse analysis	Failure index (γ)
				tonf-s^2^/m	tonf	(CCR)	(ICR)	Accept or Reject	
3-story	LAS	R	0.129	295.4	373.8	0	0	Accept	0.39
		TI 1.25	0.129	303.2	383.7	0	0	Accept	0.48
		TI 1.45	0.129	312.6	395.6	0	0	Accept	0.56
		TI 1.58	0.129	320.8	405.9	0	0	Accept	0.61
	CS	R	0.075	294.3	216.5	0	0	Accept	0.23
		TI 1.25	0.075	300.2	220.9	0	0	Accept	0.28
		TI 1.32	0.075	308.9	227.3	0	0	Accept	0.3
		TI 1.40	0.075	315.2	231.9	0	0	Accept	0.32
	GS	R	0.056	293.9	161.5	2	0	Reject	0.17
		TI 1.23	0.056	299.8	164.7	2	0	Reject	0.21
		TI 1.33	0.056	308.3	169.4	2	6	Reject	0.22
		TI 1.38	0.056	314.7	172.9	0	0	Accept	0.23
6-story	LAS	R	0.074	600.2	435.7	0	0	Accept	0.44
		TI 1.26	0.074	614.8	446.3	0	0	Accept	0.56
		TI 1.45	0.074	659.2	478.5	0	0	Accept	0.64
		TI 1.58	0.074	671.1	487.2	0	0	Accept	0.7
	CS	R	0.043	591.2	249.4	0	0	Accept	0.26
		TI 1.25	0.043	603	254.4	0	0	Accept	0.32
		TI 1.42	0.043	643.5	271.4	0	0	Accept	0.37
		TI 1.46	0.043	656.3	276.8	0	0	Accept	0.38
	GS	R	0.032	589.2	185	5	20	Reject	0.19
		TI 1.25	0.032	600.5	188.5	6	12	Reject	0.24
		TI 1.36	0.032	640.3	201	3	0	Reject	0.26
		TI 1.40	0.032	654.7	205.5	0	0	Accept	0.27
9-story	LAS	R	0.054	902.9	478.3	0	0	Accept	0.49
		TI 1.26	0.054	922.5	488.7	0	0	Accept	0.61
		TI 1.45	0.054	972.8	515.3	0	0	Accept	0.7
		TI 1.60	0.054	992.5	525.8	0	0	Accept	0.78
	CS	R	0.031	888.9	270.3	0	0	Accept	0.28
		TI 1.27	0.031	905.9	275.5	0	0	Accept	0.35
		TI 1.49	0.031	955.9	290.7	0	0	Accept	0.42
		TI 1.53	0.031	975.5	296.7	0	0	Accept	0.43
	GS	R	0.023	885.7	199.8	9	19	Reject	0.21
		TI 1.24	0.023	903.3	203.8	0	8	Reject	0.26
		TI 1.33	0.023	951.6	214.7	0	0	Accept	0.28
		TI 1.43	0.023	971.3	219.1	0	0	Accept	0.3

**Fig. 8:**
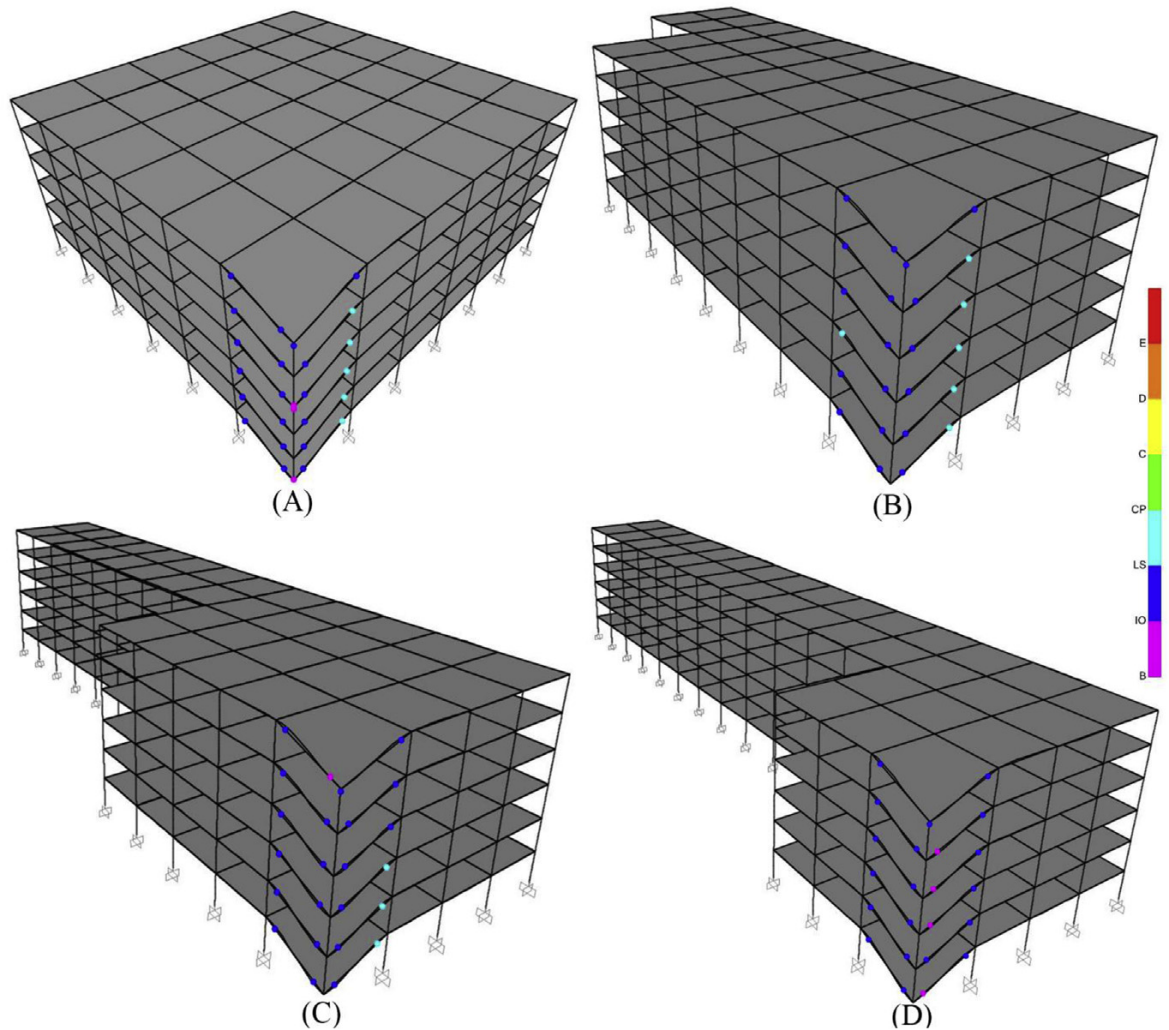
Graphical depiction of the performance of plastic hinges in the 6-story buildings with TI after progressive collapse analyses (A) R-GS (B) TI 1.25-GS (C) TI 1.36-GS (D) TI 1.4-GS.

If the value of γ is greater than 0.26, the building will have an acceptable performance after progressive failure. Although, there are two γ values of 0.23 that the buildings have acceptable performance in this Table, some γ values of 0.26 exist that have rejected condition, so the value of 0.26 is conservatively selected for discrimination point between acceptance and rejection of structure due progressive collapse. Of course, the accuracy of the above equation needs much more data, and a more accurate relation should be provided in a more comprehensive research, but this equation with its current form also gives the researcher an interesting decision-making criterion. Fig. 8 shows the plastic hinges of 6-story buildings located in the site with low seismicity after progressive failure assuming the scenario of corner column removal. According to this figure, it is observed that by increase in TI intensity, the performance level of hinges upgrades, and thereby more irregular building shows more resistance to progressive collapse.

Table 5 indicates the demand to capacity ratios (D/C) of the most critical column next to the lost column before and after the destructive event. By evaluating the D/C ratios, it is determined that increase in D/C ratio in 3-story buildings is greater than those of other taller buildings. In other words, shorter buildings are more vulnerable in the issue of increasing D/C. In addition, the D/C ratio of the column next to the removed column in a building located in the site with low seismicity level is always greater than that in a building located in the site with high seismicity level, and this shows greater backup capacity of columns of buildings located in seismic areas. Based on Table 5, again it is evident that no accurate comment can be stated concerning the scenario of removing internal or external columns and both scenarios in a progressive collapse problem should be evaluated.

**Table 5:** D/C of the most critical column before and after the progressive collapse analysis of structures with and without TI.

Number of floors	Type of site	Building	D/C before analysis	D/C after analysis	Percent of increase in D/C	D/C before analysis	D/C after analysis	Percent of increase in D/C
			(CCR)	(CCR)	%	(ICR)	(ICR)	%
3-story	LAS	R	0.28	0.54	92	0.28	0.74	164
		TI 1.25	0.25	0.54	114	0.25	0.73	192
		TI 1.45	0.25	0.53	112	0.25	0.67	170
		TI 1.58	0.19	0.36	90	0.19	0.49	161
	CS	R	0.13	0.53	295	0.27	0.62	130
		TI 1.25	0.29	0.6	107	0.34	0.78	130
		TI 1.32	0.29	0.56	94	0.34	0.78	128
		TI 1.40	0.29	0.57	99	0.31	0.84	171
	GS	R	0.35	0.64	84	0.35	0.82	137
		TI 1.23	0.3	0.57	93	0.3	0.64	117
		TI 1.33	0.3	0.57	88	0.29	0.7	140
		TI 1.38	0.29	0.52	78	0.29	0.64	117
6-story	LAS	R	0.32	0.56	78	0.32	0.7	122
		TI 1.26	0.29	0.47	60	0.33	0.55	65
		TI 1.45	0.28	0.46	62	0.33	0.55	66
		TI 1.58	0.28	0.46	62	0.33	0.56	72
	CS	R	0.54	0.74	36	0.54	0.9	66
		TI 1.25	0.53	0.79	48	0.53	0.91	71
		TI 1.42	0.52	0.81	57	0.52	0.97	89
		TI 1.46	0.52	0.79	54	0.52	0.97	87
	GS	R	0.55	0.82	49	0.55	0.93	69
		TI 1.25	0.54	0.81	50	0.54	0.89	64
		TI 1.36	0.55	0.82	50	0.56	0.87	55
		TI 1.40	0.54	0.74	36	0.56	0.87	54
9-story	LAS	R	0.41	0.64	58	0.49	0.75	51
		TI 1.26	0.4	0.66	64	0.5	0.81	63
		TI 1.45	0.39	0.71	80	0.5	0.89	78
		TI 1.60	0.4	0.68	72	0.5	0.84	68
	CS	R	0.57	0.84	48	0.65	0.98	50
		TI 1.27	0.57	0.84	48	0.54	0.91	67
		TI 1.49	0.56	0.83	47	0.56	0.93	66
		TI 1.53	0.47	0.66	39	0.54	0.79	46
	GS	R	0.57	0.87	53	0.65	0.93	42
		TI 1.24	0.58	0.8	38	0.65	0.96	48
		TI 1.33	0.57	0.83	45	0.65	0.98	50
		TI 1.43	0.58	0.79	37	0.65	0.96	47

### 4.2 Progressive collapse analyses of buildings with IDVLI

**Fig. 9:**
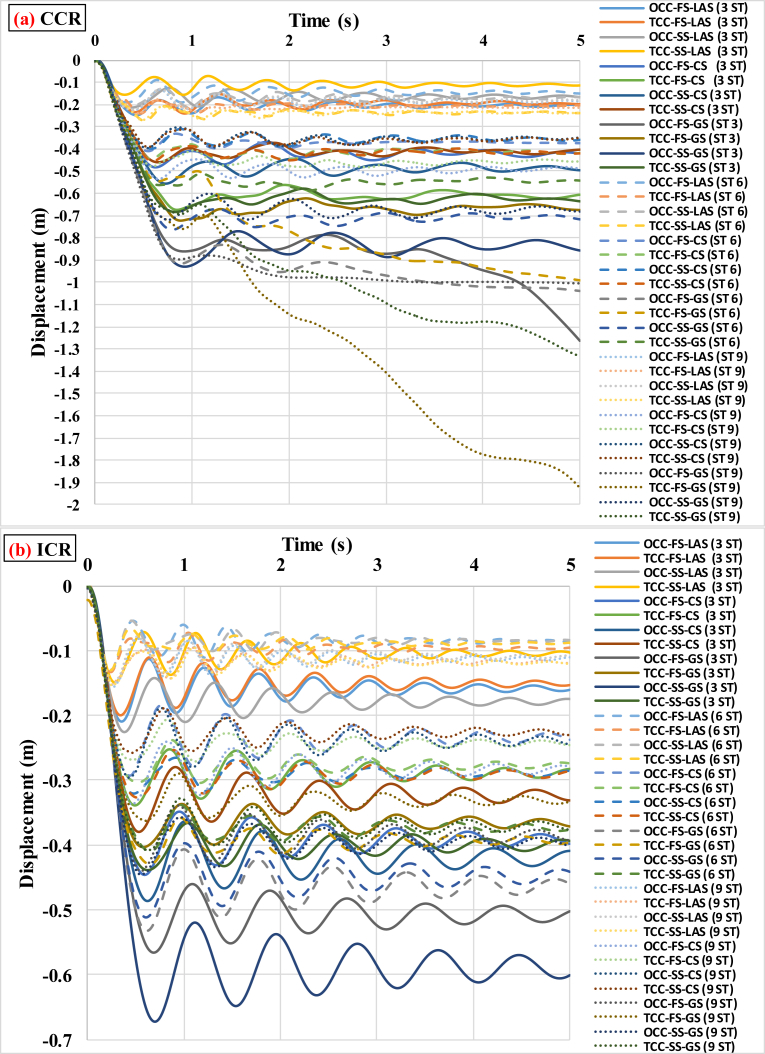
Vertical displacement histories of the 3-story buildings with IDVLI due to column cut-off under the column removal scenarios of (a) CCR and (b) ICR.

Table 6 summarizes the information obtained from progressive failure analyses under discontinuity in lateral force-resisting system in the buildings. As it is observed in Table 6, for all buildings located in different seismic sites and number of stories, the value of vertical displacement in the scenario of removing the external column is greater than that in the scenario of removing the internal column. In fact, unlike the case of TI, there is an accurate pattern for the criticality of scenarios of removing external and internal columns. On the other hand, in buildings located in various sites with different modes of IDVLI in stories, the strength of structural members in the scenario of removing the internal column is greater than that in the scenario of removing the external column. Therefore, in the case of IDVLI in system, the progressive collapse in internal columns of a building makes less damage than that in external columns of a building. However, it should be mentioned that buildings with column cut-off in perimeter were studied and the results are limited to buildings with column cut-off in facades like hotels. In addition, Table 6 indicates that for buildings located in LAS with high seismic hazard level, the value of vertical displacement of the node at the top of the removed column is less than those for buildings located in sites with medium hazard level (CS) and low hazard level (GS). Hence, it can be concluded that the seismic hazard level in the case of the IDVLI, like TI, has an important role in decrease or increase of progressive collapse potential in buildings. According to Table 6, more interesting results can be achieved that are discussed based on the scenario of removing columns in the buildings. In the scenario of removing the internal column, in the 3-story buildings located in the site with low hazard level (GS), the most severe progressive collapse occurred in the building without a column in the second story compared with the other modes assumed for this type of irregularity. Also, in the 6-story building with two columns cut-off in the first story and the 9-story building with one column cut-off in the first story located in the site with low hazard level (GS), the most severe progressive collapse was observed compared with the other modes assumed for this type of irregularity. In the scenario of removing corner column, in the 3-, 6- and 9-story buildings located in the site with low hazard level (GS), the 3- and 6-story buildings with one column cut-off and the 9-story building with two columns cut-off in the first story have the most severe progressive collapse compared with the other modes assumed for this type of irregularity. According to the results, it can be mentioned that for buildings that experience the IDVLI in height located in the site with low hazard level (GS), given the scenario of removing the internal column, the amount of damage in stories cannot be predicted, and the progressive collapse potential must be separately evaluated in each intended case. Of course, given that the scenario of removing the external column is a more critical condition, therefore considering the scenario of removing the external column is more useful and determinative for evaluation. In the scenarios of removing external columns, regardless of which story has column cut-off and the number of columns cut-off in story, the progressive collapse can be predicted in a manner that progressive collapse potential in all the buildings located in GS (low hazard level) and CS (medium hazard level) with column cut-off in the first story is higher. However, in the site with high seismicity (LAS) most of the buildings with column cut-off in the second story have a more critical condition. This indicates that buildings with more lateral resistance to earthquake with open spaces due to column cut-off in second story have a more critical condition in terms of progressive failure. With thorough evaluation of Table 6, it is revealed that the buildings with performance level of IO have the ratio of vertical displacement to span length (L) of (L/75) to (L/60). In addition, this ratio is in the range of (L/60) to (L/12) and (L/15) to (L/3) for the LS and CP performance level of buildings, respectively. These ranges are observed regardless of height and seismicity level of building. The upper limits of IO and LS limit states of aforementioned ranges are similar to the acquired limits of TI studies and the lower limits of entrance to CP performance level for both TI and IDVLI do not have significant difference. These patterns can give applicable criteria for prediction of vertical displacement of node at the top of destructed column in progressive collapse phenomenon. Indeed, the greater study with larger number of buildings can explore patterns that are more precise. Fig. 9 shows the history of vertical displacement of the node at the top of the removed column in all the buildings having the IDVLI in height in different sites.

**Table 6:** Matrix of parameters obtained from the progressive collapse analyses of the structures with IDVLI due to column cut-off.

Number of stories	Type of site	Building	T (s)	First vertical mode (ICR)	First vertical mode (CCR)	Δz (m) (ICR)	Performance level	Δz (m) (CCR)	Performance level
3-story	LAS	OCC-FS	1.26	mode = 4 T = 0.54624	mode = 4 T = 0.59201	−0.16	LS	−0.2	LS
		TCC-FS	1.287	mode = 4 T = 0.54469	mode = 4 T = 0.58142	−0.15	LS	−0.2	LS
		OCC-SS	1.275	mode = 4 T = 0.58142	mode = 4 T = 0.63301	−0.18	LS	−0.17	LS
		TCC-SS	1.234	mode = 4 T = 0.53169	mode = 4 T = 0.57762	−0.1	LS	−0.11	IO
	CS	OCC-FS	1.688	mode = 4 T = 0.7	mode = 4 T = 0.75682	−0.4	LS	−0.42	LS
		TCC-FS	1.638	mode = 4 T = 0.65556	mode = 4 T = 0.76414	−0.28	LS	−0.61	CP
		OCC-SS	1.734	mode = 4 T = 0.73637	mode = 4 T = 0.82540	−0.41	LS	−0.5	LS
		TCC-SS	1.642	mode = 4 T = 0.69812	mode = 4 T = 0.77931	−0.33	LS	−0.4	LS
	GS	OCC-FS	1.998	mode = 4 T = 0.76275	mode = 4 T = 0.91066	−0.5	LS	−1.26	CP
		TCC-FS	1.729	mode = 4 T = 0.64416	mode = 4 T = 0.74503	−0.24	LS	−0.48	LS
		OCC-SS	1.876	mode = 4 T = 0.81684	mode = 4 T = 0.91902	−0.6	CP	−0.86	CP
		TCC-SS	1.804	mode = 4 T = 0.76509	mode = 4 T = 0.86727	−0.39	CP	−0.64	CP
6-story	LAS	OCC-FS	2.129	mode = 7 T = 0.47425	mode = 7 T = 0.58007	−0.09	IO	−0.09	LS
		TCC-FS	2.094	mode = 4 T = 0.49906	mode = 4 T = 0.60609	−0.1	LS	−0.2	LS
		OCC-SS	2.113	mode = 7 T = 0.48757	mode = 7 T = 0.60696	−0.08	IO	−0.17	LS
		TCC-SS	2.129	mode = 4 T = 0.50195	mode = 4 T = 0.63112	−0.09	IO	−0.24	LS
	CS	OCC-FS	3.326	mode = 9 T = 0.62582	mode = 9 T = 0.70584	−0.24	LS	−0.37	CP
		TCC-FS	3.203	mode = 8 T = 0.64129	mode = 7 T = 0.70622	−0.27	LS	−0.42	LS
		OCC-SS	3.306	mode = 9 T = 0.65174	mode = 8 T = 0.71533	−0.29	LS	−0.36	LS
		TCC-SS	3.221	mode = 9 T = 0.65604	mode = 7 T = 0.71457	−0.29	LS	−0.42	LS
	GS	OCC-FS	3.756	mode = 9 T = 0.73814	mode = 7 T = 0.86287	−0.46	LS	−1.04	CP
		TCC-FS	3.578	mode = 8 T = 0.68950	mode = 8 T = 0.74591	−0.40	CP	−0.99	CP
		OCC-SS	3.792	mode = 9 T = 0.73966	mode = 7 T = 0.83419	−0.44	CP	−0.72	CP
		TCC-SS	3.689	mode = 8 T = 0.68851	mode = 8 T = 0.74705	−0.38	LS	−0.54	CP
9-story	LAS	OCC-FS	2.955	mode = 10 T = 0.49347	mode = 10 T = 0.59108	−0.11	LS	−0.19	LS
		TCC-FS	2.927	mode = 10 T = 0.51138	mode = 9 T = 0.58939	−0.12	LS	−0.21	CP
		OCC-SS	2.956	mode = 10 T = 0.49263	mode = 9 T = 0.59135	−0.11	LS	−0.18	LS
		TCC-SS	2.936	mode = 10 T = 0.51796	mode = 9 T = 0.61209	−0.12	LS	−0.24	LS
	CS	OCC-FS	4.9	mode = 12 T = 0.64260	mode = 10 T = 0.744	−0.28	LS	−0.49	LS
		TCC-FS	4.74	mode = 12 T = 0.61801	mode = 10 T = 0.71680	−0.25	LS	−0.46	LS
		OCC-SS	4.83	mode = 12 T = 0.61469	mode = 10 T = 0.71355	−0.25	LS	−0.35	LS
		TCC-SS	4.687	mode = 12 T = 0.61375	mode = 10 T = 0.69729	−0.23	CP	−0.35	LS
	GS	OCC-FS	6	mode = 12 T = 0.69972	mode = 10 T = 0.85594	−0.39	CP	−1	CP
		TCC-FS	5.738	mode = 12 T = 0.674	mode = 10 T = 0.80537	−0.33	LS	−1.92	CP
		OCC-SS	6	mode = 12 T = 0.70964	mode = 10 T = 0.84712	−0.4	CP	−0.68	CP
		TCC-SS	5.838	mode = 12 T = 0.69773	mode = 10 T = 0.81337	−0.38	CP	−1.33	CP

**Fig. 10:**
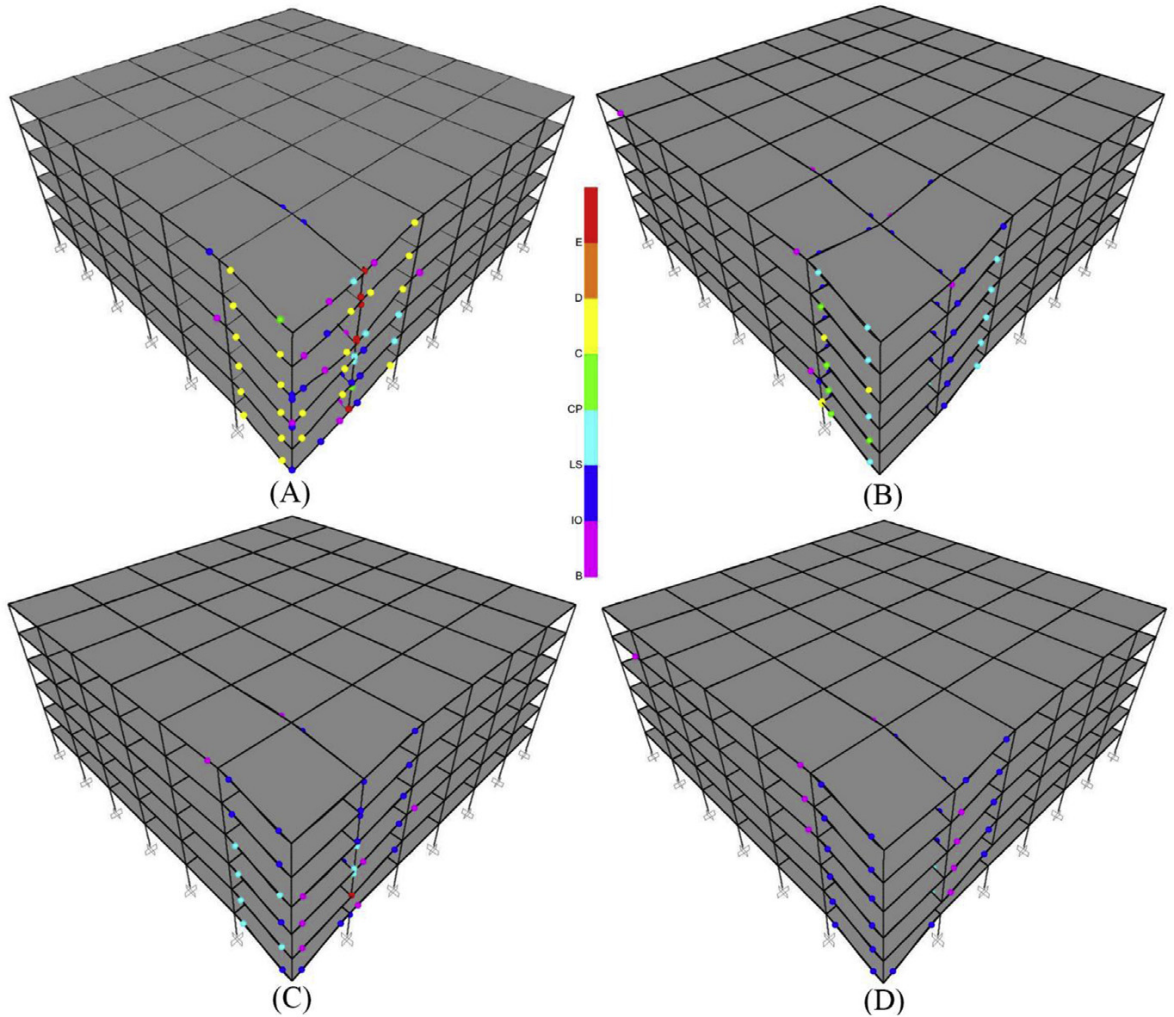
Graphical depiction of the performance of plastic hinges in the 6-story structures, designed for the GS, with IDVLI due to column cut-off after a progressive collapse analysis: A) OCC-FS, B) TCC-FS, C) OCC-SS, and D) TCC-SS.

Fig. 10 shows the plastic hinges formed in the 6-story buildings located in the site with low seismicity (GS) after progressive failure analysis. According to this figure, it is observed that one column cut-off in the first story (mode A in Fig. 10) has the highest progressive collapse potential compared to the other modes because plastic hinges have more critical conditions. In addition, Table 7 summarizes the number of hinges exceeding the CP level after progressive failure analyses. If a hinge in a building exceeds the CP level, the building is rejected, and progressive failure will lead to complete collapse. According to Table 7, removing the external column mostly creates a more critical condition in progressive failure, and the buildings located in the site with high seismicity (LAS) have been always resistant to progressive failure. Considering various scenarios of removing columns, buildings located in areas with low seismicity have always been rejected to tolerate progressive failure. Therefore, it can be concluded that buildings with void spaces and IDVLI built in areas with low seismicity do not have the required resistance to progressive failure, and some special measures should be provided for this issue in the design of these buildings.

**Table 7:** Number of plastic hinges exceeding the CP performance level and the acceptance or rejection of progressive collapse analysis for the buildings with IDVLI.

Number of stories	Type of site	Building	Base shear coefficient	Effective seismic mass	Base shear	Number of plastic hinges exceeding CP	Number of plastic hinges exceeding CP	Structural condition after progressive collapse analysis
				tonf-s^2^/m	ton	(CCR)	(ICR)	Accept or reject
3-story	LAS	OCC-FS	0.129	296.6	375.4	0	0	Accept
		TCC-FS	0.129	297.4	376.3	0	0	Accept
		OCC-SS	0.129	297	375.9	0	0	Accept
		TCC-SS	0.129	297.7	376.7	0	0	Accept
	CS	OCC-FS	0.075	294.9	217	0	0	Accept
		TCC-FS	0.075	295.4	217.4	4	0	Reject
		OCC-SS	0.075	294.7	216.8	0	0	Accept
		TCC-SS	0.075	295.1	217.1	0	0	Accept
	GS	OCC-FS	0.056	294.1	161.6	16	0	Reject
		TCC-FS	0.056	295.1	162.1	0	0	Accept
		OCC-SS	0.056	294.1	161.6	9	6	Reject
		TCC-SS	0.056	294.5	161.8	1	1	Reject
6-story	LAS	OCC-FS	0.074	601	436.3	0	0	Accept
		TCC-FS	0.074	601.8	436.9	0	0	Accept
		OCC-SS	0.074	601.4	436.6	0	0	Accept
		TCC-SS	0.074	608.1	441.5	0	0	Accept
	CS	OCC-FS	0.043	592.6	250	5	0	Reject
		TCC-FS	0.043	593.4	250.3	0	0	Accept
		OCC-SS	0.043	592.5	249.9	0	0	Accept
		TCC-SS	0.043	592.9	250.1	0	0	Accept
	GS	OCC-FS	0.032	590.5	185.4	35	0	Reject
		TCC-FS	0.032	591.6	185.7	20	0	Reject
		OCC-SS	0.032	590.3	185.3	10	1	Reject
		TCC-SS	0.032	591.1	185.6	5	0	Reject
9-story	LAS	OCC-FS	0.054	903	478.4	0	0	Accept
		TCC-FS	0.054	904.3	479.1	3	0	Reject
		OCC-SS	0.054	903	478.3	0	0	Accept
		TCC-SS	0.054	903.6	478.7	0	0	Accept
	CS	OCC-FS	0.031	890.5	270.8	0	0	Accept
		TCC-FS	0.031	891.8	271.2	0	0	Accept
		OCC-SS	0.031	890.9	270.9	0	0	Accept
		TCC-SS	0.031	892.1	271.3	0	1	Reject
	GS	OCC-FS	0.023	887.1	200.2	38	1	Reject
		TCC-FS	0.023	888.5	200.5	126	0	Reject
		OCC-SS	0.023	886.9	200.1	2	2	Reject
		TCC-SS	0.023	888.3	200.4	79	1	Reject

Table 8 indicates the demand to capacity (D/C) ratio of the most critical columns next to the lost column before and after the destructive event in the buildings with IDVLI in height. By evaluating D/C ratios, it is determined that the highest increase in D/C ratio next to lost column after the event has reached to 643%. However, in some cases, it is possible that this increase of D/C ratio still does not lead to the destruction of the column because the corresponding D/C ratio is still less than 1.0. In the 6-story buildings located in GS, after removal of column, D/C ratios have exceeded 1.0 in most of cases. Therefore, in these scenarios the values of forces can increase almost up to 6.5 times in irregular buildings with IDVLI in height. In fact, the stress level after column removal in stronger buildings located in sites with high seismicity level is often more far from failure condition due to the higher resistance of the building. This indicates more backup capacity in columns of buildings located in high seismic areas. Another important point concluded from Table 8 is that increase in the D/C ratio of columns next to lost columns is always greater in the scenarios of removing the external column, and this means that the probability of failure is greater in the scenario of external column removal. Another significant point in the behavior of buildings is that most of buildings with two columns cut-off have less increase in D/C ratio than those with one column cut-off. In other words, when the void space within the building is greater, columns around the void space have high strength, and the value of stress increase in columns next to the lost column is less in this mode. This behavior is mostly observed in the scenario of removing the external column.

**Table 8:** D/C of the most critical column before and after the progressive collapse analysis of the structures with IDVLI.

Number of stories	Type of site	Building	D/C before analysis	D/C after analysis	Percent of increase in D/C	D/C before analysis	D/C after analysis	Percent of increase in D/C
			(CCR)	(CCR)	%	(ICR)	(ICR)	%
3-story	LAS	OCC-FS	0.21	0.5	133	0.23	0.4	75
		TCC-FS	0.21	0.49	130	0.24	0.42	78
		OCC-SS	0.38	1.22	222	0.38	0.56	48
		TCC-SS	0.24	0.5	107	0.18	0.55	213
	CS	OCC-FS	0.38	0.73	90	0.29	0.55	87
		TCC-FS	0.44	1.05	137	0.51	0.68	33
		OCC-SS	0.48	1.21	153	0.48	0.79	64
		TCC-SS	0.36	0.96	171	0.36	0.96	169
	GS	OCC-FS	0.47	1.62	248	0.28	1.04	270
		TCC-FS	0.345	1	188	0.48	0.95	98
		OCC-SS	0.41	1.34	225	0.4	1.12	182
		TCC-SS	0.5	1.29	155	0.32	1.07	233
6-story	LAS	OCC-FS	0.35	1.018	188.5	0.32	0.68	111
		TCC-FS	0.49	0.69	41.5	0.32	0.72	121
		OCC-SS	0.41	1.12	175	0.39	0.78	101
		TCC-SS	0.39	1.08	180	0.23	0.89	284
	CS	OCC-FS	0.39	1.28	232	0.55	1.15	108
		TCC-FS	0.62	0.99	60.4	0.59	1.07	81
		OCC-SS	0.56	1.09	95	0.37	1.15	208
		TCC-SS	0.58	0.97	66.6	0.37	1.16	214
	GS	OCC-FS	0.25	1.82	643	0.54	0.94	74
		TCC-FS	0.6	1.47	146	0.6	1.24	108
		OCC-SS	0.58	1.13	94	0.57	1.15	102
		TCC-SS	0.4	0.98	146	0.4	1.1	178
9-story	LAS	OCC-FS	0.31	1.25	298	0.62	0.78	26
		TCC-FS	0.79	1.07	34.9	0.76	1.18	55
		OCC-SS	0.54	0.92	71	0.31	1.03	234
		TCC-SS	0.59	0.82	38.8	0.31	1.13	267
	CS	OCC-FS	0.34	1.49	339	0.59	0.94	60
		TCC-FS	0.64	1.11	73.7	0.66	0.97	46
		OCC-SS	0.37	0.84	124	0.37	1.04	179
		TCC-SS	0.36	1	180	0.36	1.13	217
	GS	OCC-FS	0.67	1.29	91.9	0.6	1	68
		TCC-FS	0.69	2.05	196	0.65	1.05	62
		OCC-SS	0.52	1.02	96	0.41	1.07	162
		TCC-SS	0.39	1.37	251	0.39	1.1	180

In this research, the effects of building height, seismicity level of site and severity of building irregularity on the progressive collapse potential were evaluated together, while in some references [17] only the parameters of the seismicity level of site and building height were discussed. Furthermore, this paper proved that both the parameters of resistance to earthquake and irregularity of structures have positive effects on mitigating progressive collapse potential, and these parameters should be evaluated together. Because each building in everywhere has its own site seismicity level and irregularity specification and considering only one parameter from the two results in an incomplete evaluation. Nevertheless, many references [12, 16, 17, 18] evaluated only one parameter solely. Meanwhile, finding of this research in contrast with some references [18, 19] proved that irregularity of building increases the resistance to progressive collapse phenomenon, because in this research equivalent-designed buildings with and without irregularity were compared.

## 5 Conclusions

Since the irregularity and seismic resistance of buildings have important effects on progressive collapse potential, in this paper, progressive collapse studies on buildings that experience TI and IDVLI have been conducted. More than 144 nonlinear dynamic analyses of progressive collapse potential on 72 buildings were carried out assuming the scenarios of removing internal and external columns. These buildings have 3, 6 and 9 stories in height, various intensities of TI and various modes of IDVLI in height, and are located in different sites in terms of seismic hazard level. The buildings with the same height were designed for almost same base shears based on relevant codes and are almost equivalent. The following results were obtained from the research:

1. In the mode of presence of TI, both scenarios of removing internal and external columns must be controlled in irregular buildings, and there is no accurate pattern for selecting the critical column removal scenario. Both the seismic hazard level of site and the TI intensity are effective on the selection of critical scenario, and no specific border can be determined for selecting the scenario of removing internal or external column.
2. Buildings with TI that are constructed in a site with high seismic hazard level (such as LAS) have more resistance to progressive collapse than those constructed in sites with low seismic hazard level (such as GS), because the strengths of beam and column sections are greater in the former case. Therefore, in cities with low seismicity, there is more concern about progressive collapse in buildings.
3. By increase in TI intensity, the progressive collapse potential in buildings decreases, because increase in TI intensity in special moment-resisting frame system leads to increase in section dimensions of beams and columns. This concept must be assessed with equivalent regular and irregular buildings in terms of equivalent base shear in order to be able to understand the result.
4. Increase in the number of stories in torsionally irregular buildings has a significant role in the reduction of progressive collapse potential so that in 6- and 9-story buildings, lower vertical displacements than that of corresponding 3-story buildings were obtained due to column removal. In other words, the severity of progressive failure was reduced in taller torsionally irregular buildings.
5. The amount of demand in plastic hinges formed due to progressive collapse is reduced by increase in design base shear coefficient, height of building and intensity of TI in a manner that all the three parameters have decisive role in collapse index of building. In this regard, a collapse index (γ) was defined and a lower limit of 0.26 for collapse in progressive failure was introduced for it. It should be noted that greater research is needed for further validation of the introduced collapse index.
6. In the mode of presence of IDVLI in height, there is an accurate pattern for the criticality of the scenarios of removing external and internal columns of buildings that have column cut-offs in facades or perimeter. In fact, the scenario of removing the external column is always more critical. In sites with different seismic hazard levels, higher strength of structural members in the scenario of removing the internal column compared to the scenario of removing the external column was observed. Hence, it can be stated that in the time of destruction in internal columns of a building, the amount of damage due to progressive failure is much less than that of destruction in external columns of the building. In buildings that have column cut-offs in interior parts, another research is needed to make comprehensive conclusions.
7. In the mode of presence of IDVLI in height, all the buildings located in the site with high seismicity (LAS) were accepted against the progressive failure phenomenon. Mutually, all the buildings located in the site with low seismicity (GS) were rejected against the progressive failure phenomenon. The buildings located in the site with moderate seismicity had a combination of acceptance and rejection and there was no accurate behavioral pattern for them.
8. Stresses of columns in building systems with architectural openings can increase up to 6.5 times due to progressive failure, which will lead to the destruction of the building. It is required to provide some special requirements for strengthening of this type of buildings against progressive failure phenomenon.
9. Buildings with IDVLI in height have a complicated behavior against progressive failure, and no accurate pattern was obtained for the effect of building height and the existence of opening in first or second story.

## Declarations

### Author contribution statement

Hamed Yavari, Mohammad Soheil Ghobadi, Mansoor Yakhchalian: Conceived and designed the analysis; Analyzed and interpreted the data; Contributed analysis tools or data; Wrote the paper.

### Funding statement

This research did not receive any specific grant from funding agencies in the public, commercial, or not-for-profit sectors.

### Competing interest statement

The authors declare no conflict of interest.

### Additional information

No additional information is available for this paper.

## Appendix A Supplementary data

Deisgnid profile section of buldings Deisgnid profile section of buldings
